# Increasing inequalities in disability-free life expectancy among older adults in Sweden 2002–2014

**DOI:** 10.1177/14034948211062309

**Published:** 2021-12-29

**Authors:** Louise Sundberg, Neda Agahi, Jonas W. Wastesson, Johan Fritzell, Stefan Fors

**Affiliations:** 1 Aging Research Center, Karolinska Institutet and Stockholm University, Stockholm, Sweden; 2 Department of Medical Epidemiology and Biostatistics, Karolinska Institutet, Stockholm, Sweden; 3 Centre for Epidemiology and Community Medicine, Region Stockholm, Stockholm, Sweden

**Keywords:** Life expectancy, mortality, morbidity, ageing, education

## Abstract

**Background:**

In an aging society with increasing old age life expectancy, it has become increasingly important to monitor the health development in the population. This paper combines information on mortality and disability and explores educational inequalities in disability-free life expectancy in the aging population in Sweden, and to what extent these inequalities have increased or decreased over time.

**Methods:**

A random sample of the Swedish population aged 77 years and above (*n*=2895) provided information about disability in the population in the years 2002, 2004, 2011 and 2014. The prevalence of disability was assessed by five items of personal activities of daily living and incorporated in period life tables for the corresponding years, using the Sullivan method. The analyses were stratified by sex and educational attainment. Estimates at ages 77 and 85 years are presented.

**Results:**

Disability-free life expectancy at age 77 years increased more than total life expectancy for all except men with lower education. Women with higher education had a 2.7-year increase and women with lower education a 1.6-year increase. The corresponding numbers for men were 2.0 and 0.8 years. The educational gap in disability-free life expectancy increased by 1.2 years at age 77 years for both men and women.

**Conclusions:**

**While most of the increase in life expectancy was years free from disability, men with lower education had an increase of years with disability. The educational differences prevailed and increased over the period as the gains in disability-free life expectancy were smaller among those with lower education.**

## Introduction

With the demographic change towards an aging society, entailing both absolute and relative increases of older adults in the population, as well as increasing life expectancy, it has become increasingly important to monitor the health development in the older population. The increase in life expectancy we are seeing now in high-income countries is mainly the result of reduced mortality among the already old [1], thus monitoring both change in mortality and health status at old age is of public health relevance. Health expectancy is a frequently used summary measure of population health that entails both the life expectancy and health status of a population. It is used for assessing several different outcomes, in which disability-free life expectancy is the most frequently used, which divides life expectancy into years with and without disability. Disability-free life expectancy has several useful features because it holds information about both quantity and quality of life and can easily be used to compare both absolute (years) and relative (proportion of life) differences between groups in the population. It can also be used to assess the changes over time and indicate to what extent increasing life expectancy is accompanied by an increase or decrease of disability in the population. There are three main hypotheses about increasing life expectancy and the presence of disability in the population. These theories are not explicit for disability, but the reasoning behind them is applicable for many health outcomes, including disability. The first hypothesis, expansion of health problems, suggests that life expectancy increases in the population mainly due to increased survival among those with health problems, while the presence and onset of these health problems remain unchanged [2]. The compression hypothesis, however, assumes that health problems can be prevented or delayed to a greater extent than the mortality decrease, and hence a compression of health problems will occur in the population [3]. The third hypothesis, the dynamic equilibrium, suggests that an increase of health problems will occur in the population. Initially, these health problems will, however, be mild and manageable and the onset of severe health problems will be delayed [4]. Although a comprehensive number of studies have addressed these hypotheses, the results are mixed, probably due to varying methodology and sampling that make comparisons of study results difficult, but also because of using different health outcomes. Overall, it appears that many high- income countries have witnessed a compression of disabilities but an expansion of chronic diseases [5].

There is a vast amount of literature on disability-free life expectancy across different settings and time periods. Many studies have explored gender differences and time trends to see if the increases in life expectancy mainly consist of years with or without disability. That is, whether there has been a compression or expansion in disability. However, disability and mortality are not evenly distributed in the population. Socioeconomic inequalities in health are widely documented and persist into old age, both in terms of disability [6] and mortality [7,8], although there are exceptions [9]. Disability-free life expectancy serves as a useful indicator to monitor socioeconomic inequality in a population, because it captures both the differences in disability and mortality. Studies of socioeconomic inequalities in disability-free life expectancy show that inequalities in disability-free life expectancy tend to be greater than inequalities in life expectancy [10]. Studies of trends in socioeconomic inequalities in disability-free life expectancy are, however, fewer, but these studies show that socioeconomic inequalities in disability-free life expectancy persist [11] or increase over time [12,13].

No studies have addressed the extent of inequalities in disability-free life expectancy or the time trend in Sweden. Other Nordic countries have pointed towards increasing inequalities in disability-free life expectancy [13] but also persistent trends [11]. Given the similarities between Nordic countries in terms of aging demographics and welfare systems, it is of interest to explore the patterns in the Swedish context in relation to the Nordic countries, but also in an international context. In this study we analyse how mortality and disability are distributed between educational groups in Sweden. We do this by using nationally representative survey data based on a random sample of the oldest old in Sweden, with high response rates and which includes both community-dwelling individuals and individuals living in care homes. The study also includes information on individuals who were not able to participate in the study themselves (e.g. due to dementia), gathered through interviews with relatives and caregivers. These are groups that are often omitted from studies of health in old age, which may result in biased estimates [14]. We use activities of daily living (ADL) for assessing disability, and education as an indicator of socioeconomic position. ADL disability is a useful measure because it captures both the independence/dependence of the individual, which is an important indicator of quality of life. It also serves as an indicator for the need for long-term care [15], which is an important aspect for resource allocation and policy. Education has the advantage of being less subjected to reverse causation with health than other socioeconomic measures, given that the educational level is established at an early stage of life [12]. There are few trend studies of health expectancies that have used these two measures in combination, and no recent study including the oldest old and individuals living in care homes.

### Aims

The aim of this study is twofold: (a) to explore remaining disability-free life expectancies at ages 77 and 85 years by educational level in Sweden in 2002, 2004, 2011 and 2014; (b) to examine if educational inequalities in disability-free life expectancies increased or decreased during the study period.

## Methods

### Study population

The study is based on two different sources of data. The age, year and education-specific prevalence of disability is gathered from the Swedish Panel Study of Living Conditions of the Oldest Old (SWEOLD) [16]. SWEOLD is based on random samples of older people (69+ years) in Sweden. It is a panel survey, with refreshments, which allows for repeated cross-sectional analyses. We used SWEOLD data from the years 2002 (net *n*=736, response rate 84.4%, age span 77–99 years), 2004 (net *n*=1352, response rate 87.3%, age span 69–100 years), 2011 (net *n*=1080, response rate 86.2%, age span 76–101 years) and 2014 (net *n*=1539, response rate 84.3%, age span 70–105 years). For consistency across waves, we used age 77 years as the lower limit, hence the total number (excluding non-response) was 2895. SWEOLD includes individuals living in care homes, and indirect interviews (proxy interviews with relatives or caregivers) were carried out when necessary. The interviews were undertaken using both face-to-face interviews (2002 and 2011) and telephone interviews (2004 and 2014). For respondents unable or unwilling to participate in face-to-face interviews in 2002 and 2011, telephone interviews were carried out instead, and as a last option postal questionnaires were offered. Similarly, for those not able/wanting to carry out the interview by telephone in 2004 and 2014, a postal questionnaire was offered. In 2011 an additional oversampling of the oldest old (85+ years) was added to the sample (*n*=355). Those oversampled in 2011, who were still alive in 2014, were also included in the 2014 survey (*n*=89). Our secondary source of data was aggregated population statistics provided by Statistics Sweden. In our analyses, the survey data were complemented with the population statistics on the age, year and education-specific mortality rates to produce life tables. These data were provided by Statistics Sweden.

### Variables

Disability was measured based on the Katz scale [17], and was defined as requiring help with at least one of the five personal ADL, which indicates the need for help and support in everyday life. A summary index was created based on the self-assessed ability independently to: eat, get dressed, go to the toilet, get in and out of bed, and wash the hair. We use the ability to wash hair, rather than the ability to shower/bath because the question of ability to shower/bath was not asked of people living in care homes in 2002 (*n*=92). As a sensitivity check, we analysed the correlations between the two items (hair washing and showering) for the other years (2004, 2011 and 2014). It was 0.81 in 2004, 0.87 in 2011 and 2014. Hence, it is unlikely that this substitution affects the final prevalence of disability substantially. Disability was defined as needing help in at least one of the five tasks, the absence of disability (disability-free) was then defined as managing all task independently. In total, there were 53 missing values (1.8%) for disability throughout the four waves and these respondents were excluded from the analysis.

In SWEOLD, educational level was assessed by self-reports. There were 61 missing values (2.1%) for education throughout the four survey waves, these respondents were excluded from the analysis. The information on education in the life tables is based on administrative registries. As few older adults in these birth cohorts have attained higher education (only 25% of the sample in 2002), we only distinguished between primary education (up to 8 years of schooling) and further education in the analyses.

Educational information is not available from the administrative registries for the cohorts born in 1911 and earlier. Hence, there is no information about educational attainment for the individuals aged 91 years and above in the life tables for 2002, those aged 93 years and above in year 2004, and those aged 100 years and above in year 2011. For this reason, the death rates in the highest ages were based on how the proportion of deaths, and mid-year population, was distributed between educational group in the previous ages. As there are some issues of data reliability regarding individuals born outside of Sweden with educational attainment from other countries [18], the mortality data only include older people born in Sweden. However, comparisons between estimates show that there is only a very small disagreement between the life expectancy estimates based on inclusion or exclusion of foreign born individuals and it does not have an impact on the results [19].

### Statistical analysis

The prevalence of disability was extracted for each sex, educational group, year and age (single years, starting at age 77 years up until the last open-ended age group of 85+ years). Probability weights were used in order to account for the oversampling of the oldest old in the SWEOLD waves of 2011 and 2014. The prevalence of disability was added to standard period life tables using the Sullivan method [20]. Hence life expectancy was divided into years with and without disability based on the proportion of individuals with disability in each age group. Ninety-five per cent confidence intervals were calculated from the standard error of disability-free life expectancy based on prevalence [21] and significance testing (*P*<0.05) and were carried out between educational groups for each year (2002, 2004, 2011 and 2014), and for the change in estimates between years 2002 and 2014. All analyses were carried out using STATA IC 15.

## Results

### Descriptive statistics

About 40% of the SWEOLD sample consisted of men across the four survey waves (Table I). The proportion of individuals who lived in care homes varied between 9% and 17%. The proportion of interviews that were, partly or wholly, conducted with a proxy respondent varied between 15% and 23%. The majority (between 49% and 73%) of the sample had only primary education (hereinafter lower education), and it can also be noted that this proportion, as expected, decreased over time. The prevalence of disability was highest in 2002, with 31% (both sexes combined, results not shown) of the sample reporting problems with at least one ADL, and lowest in 2014, when 23% reported problems with at least one ADL. Washing hair was the task people most frequently needed help with, whereas eating was the task least people needed help with.

**Table I.** Sample characteristics SWEOLD.

	2002		2004		2011		2014	
	Men	Women	Men	Women	Men	Women	Men	Women
	*n*^a^=253	*n*^a^=368	*n*^a^=253	*n*^a^=395	*n*^a^=403	*n*^a^=501	*n*^a^=312	*n*^a^=410
	%	%	%	%	%	%	%	%
Sex	40.7	59.3	39.0	61.0	37.9	62.1	41.3	58.7
Care home	12.3	16.6	9.9	10.9	8.8	13.5	9.6	14.5
Proxy^b^	17.0	23.4	20.2	22.3	17.5	23.0	14.9	18.5
Primary education	61.7	72.7	57.3	69.8	48.7	63.1	49.5	53.3
ADL disability								
Eat	4.7	6.0	3.2	6.1	4.0	2.3	6.3	6.8
Toilet	9.5	13.9	7.5	14.0	8.4	9.5	8.0	12.2
Bed	10.7	15.0	8.7	14.2	8.1	10.7	8.9	13.6
Dress	14.6	17.2	13.4	17.3	13.5	13.7	11.9	16.9
Wash hair	18.7	36.0	19.3	30.1	14.6	26.5	15.7	23.0
ADL index	21.9	36.5	19.8	30.6	20.6	27.1	17.8	26.1

ADL: activities of daily living; SWEOLD: Swedish Panel Study of Living Conditions of the Oldest Old.

Weighted proportions.

a Unweighted number.

b Interview was, partly or wholly, done with a proxy.

### Life expectancy

Between years 2002 and 2014, the increase in life expectancy at age 77 years was greater among men than among women, and greater for those with higher education compared to those with lower education (Table II). The same pattern was observed for life expectancy at age 85 years. Between 2002 and 2014 the educational gap for men was rather stable, 1.1 years in 2002 and 1.2 years in 2014. For women, the educational gap in life expectancy increased from 1.2 years to 1.5 years. A similar pattern was observed among those aged 85 years, in which the gap increased from 0.8 to 1.0 years for men and from 1.1 to 1.3 years for women (due to rounding of decimals the difference does not add up in all cases to what can be seen in Table II).

**Table II.** Life expectancy, disability-free life expectancy and disabled life expectancy by sex, age, year and education.

Sex, age (years)	Year	LE		DFLE (95% CI)		DLE (95% CI)	
		Primary	Above primary	Primary	Above primary	Primary	Above primary
Women, 77	2002	10.6	11.8	6.7 (6.1–7.2)	7.6 (6.6–8.6)	3.9 (3.4–4.5)	4.2 (3.2–5.2)
	2004	11.0	12.6	7.5 (7.0–8.1)	8.9 (7.9–10.0)^b^	3.5 (2.9–4.1)	3.6 (2.6–4.7)
	2011	11.3	12.7	7.8 (7.3–8.4)	9.9 (9.1–10.6)^b^	3.5 (2.9–4.0)	2.9 (2.1–3.6)
	2014	11.5	12.9	8.2 (7.6–8.8)^a^	10.3(9.6–11.1)^a,b^	3.3 (2.6–3.9)	2.6 (1.8–3.4)
Change		0.9	1.1	1.6	2.7	–0.7	–1.6
85	2002	5.9	7.1	2.5 (2.0–3.1)	2.6 (1.5–3.8)	3.4 (2.9–4.0)	4.4 (3.3–5.6)
	2004	6.3	7.8	3.0 (2.4–3.7)	4.3 (3.1–5.5)	3.3 (2.7–3.9)	3.5 (2.3–4.7)
	2011	6.4	7.7	3.4 (2.9–3.8)	4.5 (3.6–5.4)^b^	3.0 (2.5–3.5)	3.2 (2.3–4.1)
	2014	6.5	7.8	3.3 (2.7–3.9)	5.3(4.4–6.3)^a,b^	3.3 (2.7–3.9)	2.5 (1.5–3.4)
Change		0.6	0.7	0.7	2.7	–0.2	–2.0
Men, 77	2002	8.4	9.6	6.5 (6.0–7.1)	7.5 (6.7–8.2)^b^	2.0 (1.4–2.5)	2.1 (1.3–2.9)
	2004	8.9	10.0	7.2 (6.6–7.7)	7.8 (7.0–8.6)	1.8 (1.2–2.3)	2.2 (1.4–2.9)
	2011	9.3	10.5	6.9 (6.2–7.6)	8.7 (8.1–9.2)^b^	2.5 (1.8–3.2)	1.9 (1.3–2.4)
	2014	9.6	10.8	7.3 (6.6–7.9)	9.5 (8.9–10.0)^a,b^	2.3 (1.6–3.0)	1.3 (0.8–1.9)^b^
Change		1.1	1.3	0.8	2.0	0.3	–0.8
85	2002	4.8	5.6	2.9 (2.3–3.6)	4.2 (3.4–5.1)^b^	1.9 (1.2–2.5)	1.3 (0.5–2.2)
	2004	5.2	6.0	3.1 (2.4–3.8)	3.4 (2.3–4.6)	2.1 (1.3–2.8)	2.5 (1.4–3.7)
	2011	5.3	6.2	3.5 (3.1–3.9)	4.7 (4.1–5.3)^b^	1.9 (1.4–2.3)	1.6 (1.0–2.1)
	2014	5.4	6.4	3.2 (2.5–3.9)	5.4 (4.8–6.0)^a,b^	2.2 (1.5–2.9)	1.0 (0.4–1.6)^b^
Change		0.6	0.8	0.2	1.2	0.4	–0.4

CI: confidence interval; DFLE: disability-free life expectancy; DLE: disabled life expectancy; LE: life expectancy.

a Significantly (*P*<0.05) different estimates between 2002 and 2014.

b Significantly (*P*<0.05) different estimates between educational groups.

### Disability-free life expectancy and disabled life expectancy

**Figure 1.**
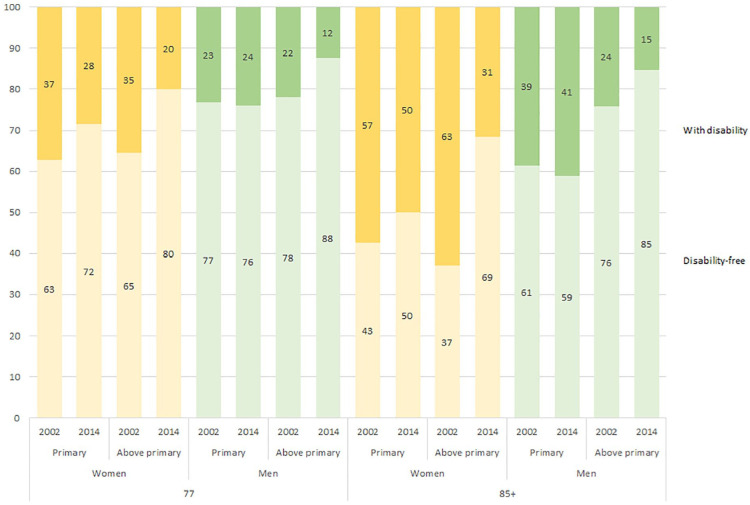
Proportion of remaining life expectancy expected to be lived with disability (top bar) and disability-free (bottom bar) by age, sex, year and education.

Table II shows disability-free life expectancy and disabled life expectancy for women at ages 77 and 85 years, for each year and for both educational groups. A more pronounced increase of disability-free life expectancy between years 2002 and 2014 was observed among those with higher education than among those with lower education, both at ages 77 and 85 years. The increase in disability-free life expectancy for women between 2002 and 2014 was statistically significant for both educational groups at age 77 years, but only for women with higher education at age 85 years. Life expectancy with disability decreased in both educational groups, but again with a more favourable development for those with higher education. In total, for those with higher education, the proportion of remaining life expectancy spent disability free increased from 65% to 80% at age 77 years, and from 37% to 69% at age 85 years. The corresponding numbers for those with lower education were 63–72% and 43–50%, respectively (Figure 1).

In concordance with the pattern observed among women, a more pronounced increase of disability-free life expectancy between years 2002 and 2014 was observed among men with higher education, both at ages 77 and 85 years. Life expectancy with disability decreased among those with higher education, while it increased for those with lower education. This pattern was observed for both those aged 77 years and those aged 85 years. However, the only statistically significant changes were the increases in disability-free life expectancy at ages 77 and 85 years among men with higher education. In total, for those with higher education, the proportion of remaining life expectancy spent disability-free increased from 78% to 88% at age 77 yearas, and from 76% to 85% at age 85 years. For those with lower education, only a minor decrease in the proportion of remaining life expectancy spent disability-free was observed, so in 2014, 76% for those aged 77 years and 59% for those aged 85 years could be expected to be lived disability free (Figure 1).

### Educational differences between 2002 and 2014

While women with both higher and lower education had an increase in disability-free life expectancy that was greater than the increase in life expectancy, this was only seen among men with higher education. For men with lower education, the increase in disability-free life expectancy did not exceed the increase in life expectancy. Comparisons between educational groups revealed that the inequalities increased over the study period. Life expectancy increased for all, but more so for those with higher education. Disability-free life expectancy also increased for all groups, but those with higher education had a greater increase. For women, the educational gap in Disability-free life expectancy at age 77 yeats increased by 1.2 years, and for women aged 85 years it increased by 2.0 years. The corresponding numbers for men were 1.2 years and 0.9 years. Life expectancy with disability decreased for all groups except for men with lower education. Hence, those with higher education experienced both a greater increase in life expectancy, and a greater increase in the time expected to live disability-free compared to those with lower education.

## Discussion

This study explored educational differences in disability-free life expectancy at ages 77 and 85 years in Sweden, between 2002 and 2014. The results showed that life expectancy increased for all educational groups between 2002 and 2014, but more so for those with higher education. Disability-free life expectancy increased more than total life expectancy during the period, except for men with lower education. Disability-free life expectancy also increased most for those with higher education. Hence, the educational inequalities in disability-free life expectancy increased during the period. However, the development was generally positive in all groups.

Having a representative sample with a high response rate when assessing the health status of an older population is important. The use of SWEOLD data in this study had the advantages of including two groups of older people that are often left out of population studies, namely those living in care homes and those who could only contribute through the help of a proxy. The inclusion of these two groups is essential for an accurate estimation of the prevalence of disability in the older population. As these groups often have poor health, excluding them leads to an underestimation of the burden of disability in the older population [14]. The high response rate of each wave of SWEOLD is also a strength of the study, as well as the standardised assessment of ADL across all four survey waves.

The study also has some limitations. The main methodological issue, which this study shares with other studies of health expectancies using the Sullivan method, is the use of prevalence data. Incidence data would have allowed the use of multistate models, which account for transitions between different disability states as well as to recovery, and thus provide more accurate estimates of disability-free life expectancies. As in many health expectancy studies based on aggregated population estimates, the mortality data used in this study are not sensitive to different mortality risks for those with and without disability. Given that those with disability have higher mortality risks [22], there is a risk of overestimating life expectancy with disability. By using prevalence data of disability there is also a risk that the disability in the population has been overestimated, because the Sullivan method assumes that disability is an absorbing state that is irreversible . However, although in reality recovery from disability is possible, the likelihood of recovery decreases with increasing age [22]. Thus, this limitation is greater in studies of younger populations. Another limitation regards the different survey modes used, as telephone interviews and face-to-face interviews have been shown to be affected differently by social desirability bias [23]. An additional limitation is the sample size of SWEOLD, which limits the statistical power to detect statistically significant differences between groups and changes over time, and limits the possibility to divide education into more than two groups. Yet, in interpreting the results we have not relied solely on whether the results are statistically significant. We have tried to weigh together the estimates, the confidence intervals and the consistency of the patterns, in order to reach reliable interpretations.

Comparisons with other studies are difficult and must be done with caution because estimates of disability-free life expectancy (and other health expectancy measures) are contingent on several assumptions and definitions. Depending on the definition of disability used (or other outcomes), different studies may yield different estimates. However, although the direct comparison between estimates is not reliable, the general trend can be compared between studies. Previous Nordic studies have shown persistent inequalities in Denmark [11] and widening inequalities in Norway [13]. This study is in line with the findings from Norway, but also other studies that have shown widening educational gaps in disability-free life expectancy [12]. Overall, we found that educational differences are larger for disability-free life expectancy than for total life expectancy, also in line with what has been found in previous studies [10]. This indicates that inequalities in disability are greater than inequalities in mortality. Moreover, those with higher education spend more years, and a larger proportion of their life, disability-free. Our results show that the difference in disability-free life expectancy between education groups increased from approximately one to two years during the study period. In a similar way, life expectancy with disability had a more favourable development for those with higher education. This pattern appears somewhat stronger among men than women, which is in line with what was found in Norway [13].

Previous studies have shown that most of the increase in life expectancy is due to reduced mortality from cardiovascular disease [24]. Reduced cardiovascular mortality has also driven much of the gains in disability-free life expectancy [25]. Those with lower education have had a greater reduction of cardiovascular mortality than those with higher education, a pattern stronger among men [26]. Yet, those with higher education have had a greater reduction in disabilities than those with lower education, as seen in this study and others [13]. This could be an indication that reduced mortality from cardiovascular disease comes at the price of a higher burden of disability for those with lower education, and that this may be especially true for men as they have had the greatest gains in life expectancy due to reductions in cardiovascular mortality.

Given what the wide field of health inequality research has shown previously, in which both morality [7, 8] and disability [6] inequality increases, our findings were not surprising. The explanation for educational inequalities in health and mortality are multifactorial [27,28]. It is possible that the change in the educational composition could have an impact on increasing inequalities, as those with the lowest education become an increasingly small and negatively selected group in terms of individual characteristics [29]. For the same reason, it has been hypothesised that the educational expansion could lead to decreasing rates of incident disabilities in the population over the upcoming decades, as groups with higher education have lower levels of disability [30].

Due to the study design that uses prevalence data and assumes equal mortality risks for people with and without disability, this study cannot conclusively settle the question of whether a compression or expansion of morbidity occurred for the oldest old in Sweden between 2002 and 2014. However, the observed patterns indicated that a compression of disability occurred for women with both high and low education, and for men with high education. For men with low education, the increase in life expectancy was still mainly composed of years without disability at age 77 years, whereas it mainly consisted of years with disability at age 85 years. Our findings show that educational differences became even more pronounced at higher ages and that the relative educational inequalities increased more for those aged 85 years.

## Conclusions

In conclusion, we found that the increasing life expectancy consists mainly of disability-free years. This suggests an overall favourable development for older adults. However, this favourable development has not been equally shared by all sections of the older population. For men with lower education the proportion of life expected to be spent with disability did not decrease over the period. Improvements in disability-free life expectancy were more pronounced among those with higher education, and as a consequence the inequalities between educational groups increased during the period.
